# Racial/ethnic differences in 21-gene recurrence score and survival among patients with estrogen receptor-positive breast cancer

**DOI:** 10.1186/s12885-024-12238-1

**Published:** 2024-04-13

**Authors:** Jasmin Gill, Keerti Yendamuri, Udit Chatterjee, Song Yao, Oluwadamilola T. Oladeru, Anurag K. Singh, Sung Jun Ma

**Affiliations:** 1 University at Buffalo, The State University of New York, 12 Capen Hall, Buffalo, NY 14260 USA; 2 Department of Radiation Medicine, Roswell Park Comprehensive Cancer Center, 665 Elm Street, Buffalo, NY 14203 USA; 3 Department of Cancer Prevention and Control, Roswell Park Comprehensive Cancer Center, 665 Elm Street, Buffalo, NY 14203 USA; 4 Department of Radiation Oncology, Mayo Clinic, 4500 San Pablo Road, Jacksonville, FL 32224 USA; 5 Department of Radiation Oncology, The Arthur G. James Cancer Hospital and Richard J. Solove Research Institute, The Ohio State University Comprehensive Cancer Center, 460 West 10th Avenue, Columbus, OH 43210 USA

**Keywords:** Oncotype, ER-positive, HR-positive, Breast CA, Racial disparities

## Abstract

**Background:**

Despite numerous studies on racial/ethnic disparities among patients with breast cancer, there is a paucity of literature evaluating racial/ethnic differences in 21-gene recurrence score (RS) and survival differences stratified by RS risk categories. We thus performed an observational cohort study to examine racial/ethnic disparities in the context of RS.

**Methods:**

The National Cancer Database (NCDB) was queried for female patients diagnosed between 2006 and 2018 with estrogen receptor (ER)-positive, pT1-3N0-1aM0 breast cancer who received surgery followed by adjuvant endocrine therapy and had RS data available. Logistic multivariable analysis (MVA) was built to evaluate variables associated with RS ≥ 26. Cox MVA was used to evaluate OS. Subgroup analyses were performed to compare the magnitude of racial/ethnic differences stratified by RS. *P* values less than 0.017 were considered statistically significant based on Bonferroni correction.

**Results:**

A total of 140,133 women were included for analysis. Of these, 115,651 (82.5%), 8,213 (5.9%), 10,814 (7.7%), and 5,455 (3.9%) were NHW, Hispanic, Black, and API women, respectively. Median (IQR) follow up was 66.2 months (48.0–89.8). Logistic MVA showed that, compared with NHW women, Black women were associated with higher RS (≥ 26 vs < 26: adjusted odds ratio [aOR] 1.19, 95% confidence interval [CI] 1.12–1.26, *p* < 0.001), while HW (aOR 0.93, 95% CI 0.86–1.00, *p* = 0.04) and API women (aOR 1.03, 95% CI 0.95–1.13, *p* = 0.45) were not. Cox MVA showed that, compared with NHW women, Black women had worse OS (adjusted hazards ratio [aHR] 1.10, 95% CI 1.02–1.19, *p* = 0.012), while HW (aHR 0.85, 95% CI 0.77–0.94, *p* = 0.001) and API (aHR 0.66, 95% CI 0.56–0.77, *p* < 0.001) women had better OS. In subgroup analysis, similar findings were noted among those with RS < 26, while only API women were associated with improved OS among others with RS ≥ 26.

**Conclusion:**

To our knowledge, this is the largest study using nationwide oncology database to suggest that Black women were associated with higher RS, while HW and API women were not. It also suggested that Black women were associated with worse OS among those with RS < 26, while API women were associated with improved OS regardless of RS when compared to NHW women.

## Introduction

Race and socioeconomic status have previously proven to be significant factors in determining an individual’s susceptibility to cancer and cancer-related survival [1, 2]. Previous studies have shown that individuals with African ancestry have a higher risk of cancer than people of other ancestral backgrounds such as European Americans and Asian Americans [3]. Among patients with breast cancer, Black women have been shown to have worse breast cancer-specific mortality than White women in a recent population-based study [4].

However, when such disparities were investigated in the context of 21-gene recurrence scores (RS), secondary analysis of major clinical trials showed no racial differences in RS [5]. This finding contradicts prior studies suggesting aggressive tumor biology and higher RS in Black women [4, 6]. Furthermore, among women with triple negative breast cancer, Black women were shown to have poor survival outcomes despite receiving comparable treatment regimens [7]. Racial differences in tumor biology may exist despite the same hormone receptor status. In addition, there is a paucity of literature evaluating survival differences stratified by RS risk groups. For example, the secondary analysis of the TAILORx trial suggested Black women with intermediate-risk RS had worse survival outcomes than White women despite receiving comparable standard-of-care treatments [5]. Racial disparity in survival outcomes may not be fully explained by differences in RS and treatments delivered. To address this knowledge gap, we performed an observational cohort study to evaluate racial/ethnic disparities in the context of RS.

## Method

Our study was approved by the institutional review board at the Roswell Park Comprehensive Cancer Center (BDR-131220). It follows the Strengthening the Reporting of Observational Studies in Epidemiology (STROBE) reporting guideline.

The National Cancer Database (NCDB) was queried for female patients diagnosed between 2006 and 2018 with estrogen receptor (ER)-positive, pT1-3N0-1aM0 breast cancer who received surgery followed by adjuvant endocrine therapy and had available RS. Racial/ethnic groups were defined as non-Hispanic White (NHW), Hispanic White (HW), Black, and Asian/Pacific Islander (API) as previously reported [1]. Other variables included for analysis were facility type, age, medical insurance, income and education level, Charlson-Deyo Comorbidity Score (CDS), year of diagnosis, histology, tumor grade, T and N staging, RS, lymphovascular space invasion (LVSI), surgery, surgical margin, radiation therapy, and chemotherapy. Lobular carcinoma represented only 12% of our entire cohort, and it was grouped together with ductal carcinoma as a common breast cancer histology for analysis. Academic centers were defined as any academic or research institution including the National Cancer Institute-designated comprehensive cancer centers. Non-academic centers included community cancer centers and integrated network cancer centers. All missing variables were coded as unknown. Other clinically relevant variables, including performance status, systemic therapy agents, and breast cancer-specific survival, were unavailable in the NCDB.

Our primary endpoint was overall survival (OS). It was defined as the time interval between diagnosis and the last follow up or death from any cause. Baseline characteristics were compared among racial/ethnic groups using Fisher exact test and Mann–Whitney U test as appropriate. Logistic multivariable analysis (MVA) was built based on baseline patient and tumor characteristics to evaluate variables associated with RS ≥ 26. Cox MVA was used to evaluate OS. OS data for patients diagnosed in 2018 were unavailable in the NCDB, and such patients were excluded for OS analysis. Cox MVA models included all variables listed previously.

Interaction term analysis was used to identify any heterogeneous association between racial/ethnic groups and RS. If the interaction term was significant, subgroup analyses were performed to compare the magnitude of racial/ethnic differences stratified by RS. To address immortal time bias due to including those treated with adjuvant endocrine therapy that might have started at different time periods, Cox MVA analyses were repeated after excluding patients with post-diagnosis survival of less than 6 months.

All *p* values were two-sided. Bonferroni correction was used for multiple comparison (NHW vs HW women, NHW vs Black women, and NHW vs API women) with *p* values less than 0.017 being statistically significant. All analyses were performed using R (version 4.0.3, R Project for Statistical Computing, Vienna, Austria).

## Results

A total of 140,133 women (median [interquartile range (IQR)] age, 60 [52–67] years) were included for analysis (Table 1). Of these, 115,651 (82.5%), 8,213 (5.9%), 10,814 (7.7%), and 5,455 (3.9%) were NHW, Hispanic, Black, and API women, respectively. Median (IQR) follow up was 66.2 months (48.0–89.8). Baseline characteristics differed significantly among racial and ethnic groups (Table 1). For example, Black women had more patients with progesterone receptor (PR)-negative tumors and grade 3 tumors with RS ≥ 26. API women had more patients with younger than 50 years of age and had private medical insurance with above median income levels.

**Table 1:** Baseline characteristics

	Non-Hispanic White (*n* = 115,651)		Hispanic White (*n* = 8213)		Black (*n* = 10,814)		Asian/Pacific Islander (*n* = 5455)		
	N	%	N	%	N	%	N	%	P
Age									< 0.001
< 50 years	20,838	18.0	2095	25.5	2309	21.4	1838	33.7	
50 years or older	94,813	82.0	6118	74.5	8505	78.6	3617	66.3	
Facility									< 0.001
Nonacademic	78,847	68.2	5108	62.2	5978	55.3	2939	53.9	
Academic	34,139	29.5	2777	33.8	4412	40.8	2225	40.8	
Not available	2665	2.3	328	4.0	424	3.9	291	5.3	
Insurance									< 0.001
None	916	0.8	349	4.2	236	2.2	98	1.8	
Private	69,729	60.3	4624	56.3	5617	51.9	3678	67.4	
Government	43,908	38.0	3129	38.1	4837	44.7	1615	29.6	
Not available	1098	0.9	111	1.4	124	1.1	64	1.2	
Income									< 0.001
Above median	69,938	60.5	4525	55.1	3767	34.8	3968	72.7	
Below median	28,332	24.5	2720	33.1	5459	50.5	769	14.1	
Not available	17,381	15.0	968	11.8	1588	14.7	718	13.2	
Education									< 0.001
Above median	66,644	57.6	3481	42.4	3076	28.4	3099	56.8	
Below median	31,800	27.5	3777	46.0	6154	56.9	1640	30.1	
Not available	17,207	14.9	955	11.6	1584	14.6	716	13.1	
CDS									< 0.001
0	98,533	85.2	6955	84.7	8276	76.5	4655	85.3	
1	13,807	11.9	1014	12.3	1953	18.1	667	12.2	
2 +	3311	2.9	244	3.0	585	5.4	133	2.4	
Year									< 0.001
2006–2013	46,201	39.9	3508	42.7	4062	37.6	1935	35.5	
2014–2017	68,843	59.5	4611	56.1	6692	61.9	3493	64.0	
Histology									< 0.001
Ductal or lobular carcinoma	99,610	86.1	7075	86.1	9145	84.6	4645	85.2	
Other	16,041	13.9	1138	13.9	1669	15.4	810	14.8	
PR									< 0.001
Positive	105,011	90.8	7481	91.1	9472	87.6	5002	91.7	
Negative	10,640	9.2	732	8.9	1342	12.4	453	8.3	
T staging									< 0.001
1	86,854	75.1	6018	73.3	7659	70.8	3889	71.3	
2	27,139	23.5	2096	25.5	2979	27.5	1510	27.7	
3	1658	1.4	99	1.2	176	1.6	56	1.0	
N staging									< 0.001
0	98,537	85.2	6965	84.8	9040	83.6	4681	85.8	
1a	17,114	14.8	1248	15.2	1774	16.4	774	14.2	
Grade									< 0.001
1	33,175	28.7	2157	26.3	2434	22.5	1320	24.2	
2	61,582	53.2	4376	53.3	5659	52.3	3071	56.3	
3	16,665	14.4	1402	17.1	2341	21.6	899	16.5	
Other	71	0.1	5	0.1	5	0.0	2	0.0	
Not available	4158	3.6	273	3.3	375	3.5	163	3.0	
RS									< 0.001
0–15	573,239	495.7	4073	49.6	4797	44.4	2685	49.2	
16–25	42,037	36.3	2943	35.8	3833	35.4	1942	35.6	
≥ 26	16,285	14.1	1197	14.6	2184	20.2	828	15.2	
LVSI									< 0.001
No	89,180	77.1	6064	73.8	8228	76.1	4149	76.1	
Yes	13,892	12.0	1075	13.1	1293	12.0	753	13.8	
Not available	12,579	10.9	1074	13.1	1293	12.0	553	10.1	
Chemotherapy									< 0.001
No	92,083	79.6	6348	77.3	8078	74.7	4181	76.6	
Yes	23,568	20.4	1865	22.7	2736	25.3	1274	23.4	
Radiation									< 0.001
No	35,409	30.6	2775	33.8	3272	30.3	1883	34.5	
Yes	79,007	68.3	5317	64.7	7393	68.4	3519	64.5	
Not available	1209	1.0	119	1.4	143	1.3	51	0.9	
Surgery									< 0.001
Lumpectomy	78,791	68.1	5374	65.4	7411	68.5	3402	62.4	
Mastectomy	36,831	31.8	2839	34.6	3400	31.4	2052	37.6	
Other	29	0.0	0	0.0	3	0.0	1	0.0	
Margin									0.01
Negative	111,871	96.7	7915	96.4	10,409	96.3	5251	96.3	
Positive	3384	2.9	270	3.3	354	3.3	178	3.3	
Not available	396	0.3	28	0.3	51	0.5	26	0.5	

*N* Number, *CDS* Charlson-Deyo Comoribidty Score, *PR* Progesterone receptor, *RS* 21-gene recurrence score, *LVSI* Lymphovascular invasion

Logistic MVA showed that, compared with NHW women, Black women were associated with higher RS (≥ 26vs < 26: adjusted odds ratio [aOR] 1.19, 95% confidence interval [CI] 1.12–1.26, *p* < 0.001), while HW (aOR 0.93, 95% CI 0.86–1.00, *p* = 0.04) and API women (aOR 1.03, 95% CI 0.95–1.13, *p* = 0.45) were not. Patients with lower income, progesterone receptor-negative tumors, LVSI, and higher tumor grades were more likely to have higher RS, while those with more recent year of diagnosis and pN1a tumors were less likely (Table 2).

**Table 2:** Multivariable logistic regression analysis of breast cancer recurrence score (RS) ≥ 26

	aOR	95% CI	P
Race			
Non-Hispanic White	Reference		
Hispanic White	0.93	0.86–1.00	0.04
Black	1.19	1.12–1.26	< 0.001
Asian/Pacific Islander	1.03	0.95–1.13	0.45
Age			
< 50 years	Reference		
50 years or older	1.05	1.00–1.10	0.06
Facility			
Nonacademic	Reference		
Academic	0.98	0.94–1.01	0.19
Insurance			
None	Reference		
Private	1.02	0.88–1.19	0.82
Government	0.97	0.84–1.14	0.74
Income			
Above median	Reference		
Below median	1.1	1.05–1.16	< 0.001
Education			
Above median	Reference		
Below median	1.04	0.99–1.08	0.12
CDS			
0	Reference		
1	1.02	0.97–1.07	0.41
2 +	1.09	0.99–1.20	0.08
Year			
For every 1 year increase	0.98	0.97–0.99	< 0.001
Histology			
Ductal or lobular carcinoma	Reference		
Other	0.74	0.70–0.78	< 0.001
PR			
Positive	Reference		
Negative	6.59	6.31–6.89	< 0.001
T staging			
1	Reference		
2	1.31	1.26–1.36	< 0.001
3	0.8	0.68–0.92	0.003
N staging			
0	Reference		
1a	0.8	0.76–0.85	< 0.001
Grade			
1	Reference		
2	2.75	2.60–2.91	< 0.001
3	16.98	16.02–18.02	< 0.001
Other	21.15	13.28–33.64	< 0.001
LVSI			
No	Reference		
Yes	1.22	1.16–1.28	< 0.001

*aOR* Adjusted odds ratio, *CI* Confidence interval, *CDS* Charlson-Deyo Comoribidty Score, *PR* progesterone receptor, *LVSI* Lymphovascular invasion

**Fig. 1:**
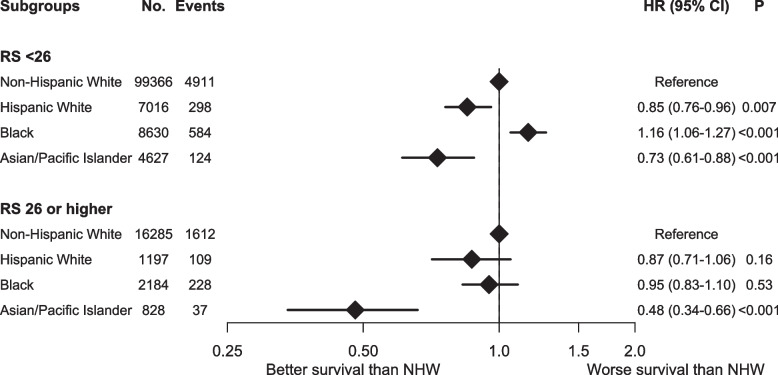
Forest plot of subgroup analyses for overall survival stratified by racial/ethnic groups and 21-gene recurrence score. Overall survival outcomes were compared stratified by different racial/ethnic subgroups and 21-gene recurrence score. No: number of patients; HR: hazards ratio; 95% CI: 95% confidence interval; RS: 21-gene recurrence score; NHW: non-Hispanic White women

Cox MVA showed that, compared with NHW women, Black women had worse OS (adjusted hazards ratio [aHR] 1.10, 95% CI 1.02–1.19, *p* = 0.012), while HW (aHR 0.85, 95% CI 0.77–0.94, *p* = 0.001) and API (aHR 0.66, 95% CI 0.56–0.77, *p* < 0.001) women had better OS (Table 3). Variables associated with worse OS were older age, non-private medical insurance, below median income and education levels, more comorbidities, PR-negative status, higher T and N staging, higher tumor grades and RS, LVSI, and positive margin (Table 3). There was a statistically significant interaction between race/ethnicity and RS (interaction *p* = 0.006). In subgroup analysis, similar findings were noted among those with RS < 26, while only API women were associated with improved OS among others with RS ≥ 26 (Fig. 1).

**Table 3:** Multivariable Cox regression analysis of overall survival

	aHR	95% CI	P
Race			
Non-Hispanic White	Reference		
Hispanic White	0.85	0.77–0.94	0.001
Black	1.1	1.02–1.19	0.01
Asian/Pacific Islander	0.66	0.56–0.77	< 0.001
Age			
< 50 years	Reference		
50 years or older	1.9	1.73–2.08	< 0.001
Facility			
Nonacademic	Reference		
Academic	0.79	0.75–0.83	< 0.001
Insurance			
None	Reference		
Private	0.62	0.51–0.76	< 0.001
Government	1.44	1.18–1.77	< 0.001
Income			
Above median	Reference		
Below median	1.17	1.11–1.25	< 0.001
Education			
Above median	Reference		
Below median	1.06	1.00–1.13	0.04
CDS			
0	Reference		
1	1.59	1.50–1.68	< 0.001
2 +	2.72	2.51–2.95	< 0.001
Year			
For every 1 year increase	1.02	1.01–1.03	0.002
Histology			
Ductal or lobular carcinoma	Reference		
Other	0.98	0.92–1.05	0.58
PR			
Positive	Reference		
Negative	1.09	1.02–1.16	0.02
T staging			
1	Reference		
2	1.49	1.42–1.57	< 0.001
3	2.26	1.95–2.63	< 0.001
N staging			
0	Reference		
1a	1.61	1.52–1.71	< 0.001
RS			
0–15	Reference		
16–25	1.16	1.10–1.22	< 0.001
≥ 26	2.05	1.91–2.21	< 0.001
Grade			
1	Reference		
2	1.14	1.08–1.21	< 0.001
3	1.5	1.39–1.61	< 0.001
Other	1.02	0.49–2.15	0.95
LVSI			
No	Reference		
Yes	1.15	1.08–1.23	< 0.001
Chemotherapy			
No	Reference		
Yes	0.7	0.65–0.74	< 0.001
Radiation			
No	Reference		
Yes	0.6	0.55–0.64	< 0.001
Surgery			
Lumpectomy	Reference		
Mastectomy	0.73	0.67–0.79	< 0.001
Other	1.47	0.55–3.94	0.44
Margin			
Negative	Reference		
Positive	1.18	1.04–1.33	0.008

*aHR* Adjusted hazards ratio, *CI* Confidence interval, *CDS* Charlson-Deyo Comoribidty Score, *PR* Progesterone receptor, *RS* 21-gene recurrence score, *LVSI* Lymphovascular invasion

After excluding those with post-diagnosis survival of less than 6 months, Black women still had worse OS (aHR 1.11, 95% CI 1.03–1.19, *p* = 0.009) compared to NHW women, while HW (aHR 0.85, 95% CI 0.77–0.94, *p* = 0.002) and API (aHR 0.65, 95% CI 0.56–0.77, *p* < 0.001) women still had better OS. The interaction between race/ethnicity and RS was also statistically significant (interaction *p* = 0.006). The findings of the subgroup analysis remained to be similar. Among those with RS < 26, Black women continued to have worse OS (aHR 1.17, 95% CI 1.07–1.28, *p* < 0.001), while HW (aHR 0.85, 95% CI 0.76–0.96, *p* = 0.008) and API (aHR 0.73, 95% CI 0.61–0.87, *p* < 0.001) women continued to have better OS. Among others with RS ≥ 26, only the association of API race with improved OS was significant (aHR 0.48, 95% CI 0.35–0.67, *p* < 0.001).

## Discussion

To our knowledge, this is the largest study using a nationwide oncology database to demonstrate that Black women had higher RS than NHW women. It also showed that Black women had worse OS among those with RS < 26, while API women had better OS regardless of RS when compared to NHW women.

Theories for these inferior outcomes in Black women include adverse tumor biology, differences in access to care, lack of appropriate adjuvant therapy, and/or adherence to treatment [8–10]. Several socioeconomic factors have been noted as having a significant impact on survival for Black women with breast cancer, including lack of access to care and suboptimal treatment [6]. However, one study performed at private community oncology practices, including patients who completed similar treatment regimens across all racial and ethnic groups, suggested that Black women still had poor survival outcomes, suggesting that tumor biology may be contributing to racial disparity in outcomes [7]. This supports findings in our study, which demonstrated that Black women may be associated with more aggressive tumor biology, including higher RS.

Our main finding of the association of Black race with higher RS in our study is consistent with a recent population-based study [4]. More aggressive tumor biology indicated by higher RS in our study supports a growing body of literature showing racial disparities in overall survival [7] and breast cancer-specific survival among patients with breast cancer [2]. Such findings were also observed among patients within intermediate-risk RS in the secondary analysis of the TAILORx trial [5]. However, in this secondary analysis, both White and Black women had comparable RS unlike findings from our study, and this discrepancy may be due to relatively smaller sample size of patients from racial minority in the TAILORx trial compared to our large cancer registry database [5]. Nonetheless, racial disparities in survival outcomes may be explained by both social determinants of health as well as aggressive tumor biology, [6] and our study further supports discrepancies in tumor biology based on racial and ethnic groups. In our current study, other adverse features correlated with higher RS were PR-negative status and tumor grade, consistent with our prior study [11] and others [12, 13]. Our study showed a higher proportion of Black women with PR-negative status and higher tumor grade, and such adverse features may be in part contributing to higher RS among Black women.

In our study, there were no statistically significant differences amongst racial groups of women with RS ≥ 26 other than API women having better survival. Although a secondary analysis of the TAILORx trial also showed no racial disparities in survival outcomes among those with RS ≥ 26, the sample size of patients with RS ≥ 26 was small and improved survival among the API women was not observed [5]. Consistent with our findings, other studies suggested that API women had the lowest incidence and death rates from breast cancer [14]. Another study also demonstrated that API women are associated with better survival than White women, independent of histology or hormone receptor status [5]. However, reasons for favorable survival among API women were unclear, since there was no statistically significant difference in RS between non-Hispanic White and API women in our study. API women are highly heterogeneous in prognosis and survival outcomes depending on their social determinants of health, such as socioeconomic status and education levels [15]. Our finding may not be applicable to all racial and ethnic subgroups among API women.

Limitations of our study include inherent biases given its retrospective nature. A number of relevant variables were unavailable for analysis, such as additional treatment interventions including re-excision after positive margin, tumor recurrence, breast cancer-specific survival, systemic therapy agents, and adherence to treatments, which may lead to residual confounding despite adjusting for other available variables. In addition, a small proportion of patients had high RS in prior population-based studies, [16, 17] and our subgroup analysis involving those with RS ≥ 26 might not be adequately powered to detect OS differences across racial groups. Furthermore, given the nature of OS as an endpoint and the low number of events available for analysis, our study was not adequately powered to investigate racial and ethnic differences in OS stratified by different age and nodal staging subgroups in addition to RS.

## Conclusion

Our observational cohort study using nationwide oncology database suggested that Black women had higher RS and further, had worse OS among those with RS < 26, while API women had improved OS regardless of RS when compared to NHW women.

## Data Availability

Data are publicly available through the American College of Surgeons (ACS) for eligible investigators affiliated with Commission on Cancer (CoC)-accredited cancer programs (https://www.facs.org/quality-programs/cancer-programs/national-cancer-database/).
